# Cilia Action in Islets: Lessons From Mouse Models

**DOI:** 10.3389/fendo.2022.922983

**Published:** 2022-06-23

**Authors:** Jung Hoon Cho, Jing W. Hughes

**Affiliations:** Department of Medicine, Washington University School of Medicine, Saint Louis, MO, United States

**Keywords:** primary cilia, β-cells, pancreatic islets, insulin secretion, hormone regulation

## Abstract

Primary cilia as a signaling organelle have garnered recent attention as a regulator of pancreatic islet function. These rod-like sensors exist on all major islet endocrine cell types and transduce a variety of external cues, while dysregulation of cilia function contributes to the development of diabetes. The complex role of islet primary cilia has been examined using genetic deletion targeting various components of cilia. In this review, we summarize experimental models for the study of islet cilia and current understanding of mechanisms of cilia regulation of islet hormone secretion. Consensus from these studies shows that pancreatic cilia perturbation can cause both endocrine and exocrine defects that are relevant to human disease. We discuss future research directions that would further elucidate cilia action in distinct groups of islet cells, including paracrine and juxtacrine regulation, GPCR signaling, and endocrine-exocrine crosstalk.

## Introduction

Primary cilia are solitary antenna-like structures that project from the cell bodies of most vertebrate cells. Cilia serve as crucial signaling organelles that integrate environmental sensing with cellular functions such as proliferation, differentiation, and energy homeostasis. Defects in primary cilia cause human disease, including metabolic disorders such as obesity and type 2 diabetes (1–3). However, the metabolic abnormalities seen in human ciliopathies such as Bardet-Biedl and Alström syndromes result from the cumulative loss of primary cilia in multiple organ systems (4–7), while targeted cilia deletion models were required to understand true pancreatic and islet cilia contribution to metabolic diseases. A number of key experimental mouse models have been developed in the past two decades, examining pancreatic and metabolic phenotypes associated with ciliary dysfunction (8–18). As the pancreatic islets are essential for the production of glucoregulatory hormones, functional studies of islet cilia have focused on β-cells where cilia appear to play a strong role in insulin secretion. These single-gene knockout mouse models mimic mutations seen in human ciliopathies and offer new angles to examine β-cell dysfunction in human diabetes in general. In accord with knockout studies, cilia-related genes have been queried in diabetes-prone obese mouse strains as well as human subjects with diabetes, showing diabetes-related changes in cilia gene expression and corresponding perturbations in cell cycle regulation, highlighting the relevance of islet cilia research to human metabolic disease (19, 20). Here, we present an update on recent studies of islet cilia using mouse models (**Table 1**) and discuss the emerging complexity of ciliary functions in glucose regulation.

**Table 1:** Mouse models used in studying pancreatic islet cilia .

Mouse model	Pancreas pathology	Obesity	Glucoseregulation	Glucagonsecretion	Insulinsecretion	Somatostatinsecretion	References
Whole body Tg737^orpk +/+^	Pancreatic mass ↓ Ductal dilation	No	-	-	-	-	Cano et al. (2004) (8)
Whole body Tg737^orpk +/+^	Acinar fibrosis Normal islet size	No	Fasting serum glucose ↓ Glucose tolerance ↓	–	–	–	Zhang et al. (2005) (9)
Whole body Alms1 KO	Islet hypertrophy	Yes	Fed & fasting serum glucose ↑ Glucose tolerance ↓	-	Fed serum insulin ↑	-	Collin et al. (2005) (10) Arsov et al. (2006) (11)
β-cell-specific Pdx1-Cre Kif3a KO/cKO	Acinar-to-ductal metaplasia Lipomatosis Ductal cysts Normal islet size	No	Normal glucose tolerance	-	-	-	Cano et al. (2006) (12)
Whole body Kif3a cKO	Not evaluated	Yes	Fasting serum glucose ↑	-	Fed serum insulin ↑	-	Davenport et al. (2007) (14)
Whole body Rfx3 KO	α-cell mass ↓ β-cell mass ↓ PP cells ↑ Ghrelin cells ↓	No	Fasting serum glucose ↑ Glucose tolerance ↓	-	*ex vivo* GSIS ↓	-	Ait-Lounis et al. (2007) (13)
β-cell-specific Pdx1-Cre LKB1 cKO	β-cell size ↑ Altered β-cell polarity	No	Normal fed & fasting serum glucose Glucose tolerance ↑	-	*ex* & *in vivo* GSIS ↑	-	Granot et al. (2009) (15)
Whole body Bbs4 KO	Normal islet size	Variable	Glucose tolerance ↓ Fasting serum glucose ↑	-	Elevated or normal fasting serum insulin	-	Eichers et al. (2006) (21) Gerdes et al. (2014) (16)
β-cell-specific	β-cell mass ↓	No	Glucose tolerance ↓	-	Fasting serum insulin ↓	-	Volta et al. (2019) (17)
Pdx1-Cre IFT88 cKO (βICKO)					*ex* & *in vivo* GSIS ↓		
β-cell-specific Ins1-Cre IFT88 KO/cKO (βCKO)	β-cell mass ↓ δ-cell mass ↑	No	Fasting serum glucose ↑ Glucose tolerance ↓	Fed serum glucagon ↑ Glucagon secretion ↑	Fed serum insulin ↓ *ex* & *in vivo* GSIS ↓	Somatostatin secretion ↑	Hughes et al. (2020) (18)

Orpk: Oak ridge polycystic kidney; Alms1: Alstrom syndrome 1; Pdx1: Pancreatic and duodenal homeobox 1; Rfx3: Regulatory factor X3; Kif3a: Kinesin family member 3A; LKB1: Liver kinase B1; Bbs4: Bardet-Biedl syndrome 4; IFT88: Intraflagellar transport 88; Ins1: Insulin 1; cKO: Conditional knockout; GSIS: Glucose-stimulated insulin secretion

↑, Increase; ↓, Decrease; -, Not evaluated.

## Pancreatic Islet Cilia Classification and Topology

Primary cilia have been identified by electron microscopy (EM) studies on both pancreatic exocrine and endocrine cells (22–26). Two main types of cilia, primary and motile cilia, are distinguished by their abundance, ultrastructural configuration, and capacity for movement. Classic primary cilia are solitary, composed of nine cylindrical outer microtubule doublets without a central pair (“9+0”), and considered non-motile. In contrast, motile cilia are multi-cilia that are generally present in tens to hundreds per cell and possess central microtubules (“9+2”) as well as other critical structural motifs including dynein arms, radial spokes, and nexin-dynein regulatory units that confer motility to the axoneme (27, 28). A third class, nodal cilia, are present in the developing embryo and possess primary cilia-like structure yet are motile, and their ciliary movements are required for left-right asymmetry specification (29). Pancreatic islet primary cilia, like the monocilia of many other organ systems, are thought to be strictly sensory with a classic “9+0” configuration, but anecdotal findings of non-”9+0” microtubule arrangements from rodent islet cilia EM studies have challenged this assumption (22–25, 30). More recently, complete 3D tomography maps of primary cilia from various cell types have revealed dynamic rearrangements of ciliary microtubules along the axoneme (31–33), suggesting that the capture of 9 + 0 or non-9+0 cilia cross-sections may be sampling-dependent and that both structures may exist within primary cilia, even within a single cilium. Consistent with these ultrastructural observations, we have recently reported on motile properties and non-9+0 configuration of human β-cell primary cilia, which depend on active dynein-ATP forces and are required for insulin secretion (34)(preprint on bioRxiv, manuscript in late-stage review). Together these new evidence preliminarily suggest that islet primary cilia may possess both sensory and motile capacity and that both functions may be important to islet hormone secretion.

β-cells comprise the largest group of endocrine cells in the islets of Langerhans and display unique cytoarchitectural arrangements that underlie their function. There exist significant differences in the cellular composition and arrangements of rodent versus human islets, but in both species, β-cell polarity and connectivity are important for insulin secretion. β-cells establish physical connections with both other β-cells and non-β-cells, including glucagon-secreting α-cells and somatostatin-secreting δ-cells. Groups of β-cells are often organized in rosette-like clusters around the islet vasculature, with their bases abutting the arteriole and the cell vertex pointing to the venous capillaries, which gives directionality to insulin secretion (35–37). This artery-to-vein axis helps to establish polarity of the β-cell and defines the apical, basal, and lateral surfaces of the β-cells as distinct functional domains (37–39). The primary cilium is preferentially located on the lateral surface of the β-cell, extending into a luminal space and toward the vascular apogee (15, 37). This intercellular lumen contains the elaborate canaliculi system where interstitial fluid flows, connecting the arterial to the venous blood vessels and likely representing an important site of glucose uptake by β-cells (40). It also provides a space for multiple primary cilia from adjacent cells to potentially interact (37) and form homotypic (β-β) and heterotypic (β-non-β) cilia connections. Therefore, the location of primary cilia in this intra-islet canaliculi space is meaningful to their function as cellular sensors, hormone regulators, and cell-cell communications devices.

## Cilia and Pancreatic Islet Development

The intraflagellar transport (IFT) complex is essential for the assembly and maintenance of all cilia, primary or motile (41). A key protein component of this complex, IFT88 (also called polaris, Tg737, and orpk), is localized throughout the ciliary basal body and axoneme and plays a key role in ciliogenesis by transporting the building blocks of the ciliary axoneme and membrane (42, 43). Loss of IFT88 causes missing cilia, manifesting most saliently as polycystic kidney disease in both mouse and humans (43, 44). Limited by the early lethality in whole-body Tg737^orpk^ mouse models, effects of homozygous IFT88 mutation on the pancreas have been mostly studied developmentally by histology in embryonic pancreata, showing reduced cilia number and aberrant cilia architecture, with simultaneous acini loss and ductal cell proliferation and cyst formation (8) (**Table 1**). An endocrine defect was revealed upon dynamic metabolic testing in young (2-week-old) homozygous IFT88 mice showing reduced tolerance of both short-term fasting and acute glucose challenge compared with age-matched wild-type mice (9). Collectively, these observations in Tg737^orpk^ mice were the first to link cilia defects to pancreatic pathology, revealing a role of primary cilia in the development and maintenance of both exocrine and endocrine compartments (8, 9).

Primary cilia formation requires transcriptional control, most prominently by the regulatory factor X (RFX) family proteins. Among these, RFX3 has important roles in directing the biogenesis of primary cilia, motile cilia, and nodal cilia, where Rfx3-deficient mice exhibit growth retardation and laterality defects (13, 45–47). In the mouse pancreas, RFX3 expression is restricted to endocrine cells during embryogenesis and in adult animals, where it regulates the expression of cilia-related genes including Dync2li1 and Ift88 (13). Rfx3 inactivation during pancreatic development causes loss of cilia and altered islet cellular composition, including smaller islet size with reduced number of α-, β-, and ghrelin-positive cells and disorganized cytoarchitecture (13) (**Table 1**). In adult mice, RFX3-dependent cilia loss leads to reduced insulin content and secretion, as well as fasting hyperglycemia and glucose intolerance (13). These findings implicated a strong role of the ciliogenesis transcription factor RFX3 in pancreatic islet development and showed that cilia formation is required for proper endocrine cell differentiation and function.

## Two Human Ciliopathies and Their Mouse Models

Human ciliopathy syndromes caused by ciliary gene mutations lead to pleiotropic clinical manifestations, including kidney disease, retinal degeneration, hearing loss, cognitive impairment, hypogonadism, and metabolic defects. In particular, Alström syndrome (ALMS) and Bardet-Biedl syndrome (BBS), two autosomal recessive disorders, exhibit prominent metabolic features including accelerated obesity, insulin resistance, and type 2 diabetes (2, 6, 7). Much of the genetic basis for both ALMS and BBS has been elucidated, and animal models have been developed to probe gene-specific disease mechanisms. ALMS is a monogenic disease strongly resembling human type 2 diabetes with severe insulin resistance (4, 7). The ALMS protein is a component of the human centrosome (48); Alms1 gene-trapped mice form normal size and number of cilia but exhibit faulty vesicle docking to the ciliary base and impaired intracellular trafficking (10). These animals develop metabolic changes including obesity, hypercholesterolemia, hyperinsulinemia, insulin resistance, and hyperglycemia. Specific to pancreatic pathology, these mice exhibit compensatory islet beta cell hyperplasia in response to insulin resistance and resultant increased demand for insulin secretion (10) (**Table 1**). Another ALMS mouse model, the *fat aussie* mouse with exon 8 Alms1 deletion, develops spontaneous diabetes with elevated fasting glucose and glucose intolerance, where even massive islet hyperplasia cannot produce enough insulin to match the demand (11). Islet function in these models, however, was not examined in isolation, independent of the whole-body metabolic defects, so it remained unclear whether Alms1-dependent ciliary changes and the diabetes phenotype seen in rodent models were related to intrinsic β-cell or islet dysfunction.

In contrast to ALMS, BBS is a genetically heterogeneous disease that is linked to loss-of-function pathogenic variants in over 26 causative genes. Many of these genes encode subunits of the BBSome protein complex, which regulates ciliary cargo trafficking (49, 50). Examinations of whole-body knockout and knockin mouse models for the genes Bbs1-8 and Bbs10 have revealed conserved phenotypes of obesity or increased fat mass, hyperphagia and decreased caloric expenditure, which likely result from a combination of neuroanatomical changes and peripheral metabolic organ defects (51–59), in addition to non-metabolic abnormalities that are not discussed herein. Among these mouse models, Bbs4-null mice have been most studied for their metabolic phenotypes, which include abnormal lipid profiles, liver dysfunction, and defects in glucose regulation (16, 21). When examined in isolation *ex vivo*, Bbs4-null islets show blunted first-phase insulin secretion, suggesting that the loss of ciliary function produces islet-intrinsic defects (**Table 1**). Intriguingly, Bbs4 mice show defects in ciliary targeting of both the insulin receptor (IR) and of the G protein-coupled receptor (GPCR) somatostatin receptor 3 (SSTR3) (16, 60), raising the possibility that, in pancreatic islets, primary cilia may regulate paracrine or autocrine signaling *via* locally secreted hormones such as insulin and somatostatin following glucose stimulation. These early models demonstrated the complexity of the potential roles of primary cilia in fine-tuning islet cell crosstalk which awaited clarification from islet cell-specific cilia knockout models.

## KIF3A as a Regulator of Pancreatic Function

The KIF3 family of proteins are key subunits of kinesin-II motors, including KIF3A which is a ciliogenic factor that has a varied role in islet function. KIF3A heterodimerizes with KIF3B and 3C to form motors that transport IFT particles along the anterograde microtubules in cilia and flagella (61). Whole-body Kif3a null mutant mice exhibit early lethality, randomized left-right asymmetry, and ciliary morphogenesis defects in embryonic nodal cilia (62). To examine the role of cilia function in adult mice, two studies generated global or tissue-specific inducible Kif3a knockout mice (12, 14) (**Table 1**). Tamoxifen-induced loss of whole-body Kif3a in adult mice led to hyperphagia and increased weight gain, a defect phenocopied by inducible whole-body IFT88 knockout mice and corrected by pair-feeding (14). These mice had elevated fasting glucose and fed insulin levels in sera, suggesting a strong defect in glucose metabolism, though due to the global knockout was not directly attributable to cilia action in the pancreas. A more specific pancreatic Kif3a deletion model was made using early and late onset Pdx1-Cre lines (63) to temporally target cilia expression in ductal progenitor cells, which revealed a number of exocrine pathologies including ductal hyperplasia and dilation and acinar cell loss, but these mice had no obvious endocrine defects and no obesity phenotype, at least within the first three months of life (12). A more recent study in MIN6 insulinoma cells and primary mouse and human islets showed that shRNA knockdown of KIF3A led to reduced ciliation and islet cell proliferation, where the absence of cilia may disrupt cell division, but short-term KIF3A gene silencing had no apparent effects on glucose-stimulated insulin secretion (19). These mixed findings regarding the role of KIF3A in pancreatic endocrine function may be related to differences in the Cre driver lines used, efficiency and timing of gene knockout/knockdown, and non-pancreatic cilia effects on metabolism. Consistent in most of these studies was a role of KIF3-mediated ciliation in exocrine pancreas development and maintenance. Taken together, these results indicate that KIF3A may be an important regulator in the exocrine pancreas, while its role in maintaining endocrine function may depend on developmental stage and other modifying factors such as cilia function in other metabolic tissues and organ systems.

## LKB1, Cilia Position, and β-Cell Polarity

An interesting link between primary cilia and β-cell polarity was revealed by a model of liver kinase B1 (LKB1) deficiency (15), which models the genetic defect in human Peutz-Jeghers syndrome, an autosomal dominant germline disease manifested by intestinal polyposis and pancreato-biliary cancers (64–66). LKB1 encodes a serine-threonine kinase that is expressed in multiple intracellular compartments and is enriched in the primary cilium, where its activity is postulated to induce AMPK phosphorylation at the cilium base to regulate metabolic signaling (67). In mouse pancreas, deletion of LKB1 in adult islets leads to a repositioning of primary cilia from the β-cell lateral surface to the opposite pole of the cell, away from the intrarosette capillary. Meanwhile, there is a corresponding change in the anatomical relationship of β-cells to blood vessels, with altered β-cell polarity where the nucleus is abnormally located at the cell base near islet capillaries, and decreased GLUT2 expression on the β-cell lateral membrane where cilia and microvilli reside (15) (**Table 1**). Functionally, consistent with its role as a tumor suppressor, LKB1 inactivation in islets led to increased β-cell size and increased insulin secretion. While there remain questions regarding the mechanism of cell polarity shifts and potential LKB1-dependent signaling in the β-cell cilium, these findings do suggest that β-cell polarity and in particular β-cell cilia polarity may be linked to cellular energy sensing and cell growth decisions.

## Targeted IFT88 Deletions Reveal Cilia Roles in β-Cell Function

The interpretation of early IFT88 models was limited by the lack of pancreas- and cell-type specificity in their cilia gene deletion. To directly test the role of IFT88 and of primary cilia in β-cell function, two cell-specific models of IFT88 deletion have been generated, showing comparable results (**Table 1**). These include the inducible and constitutive cilia knockout βICKO and βCKO mice that ablated IFT88 expression under the control of Pdx1-CreER and Ins1-Cre, respectively (17, 18). Both Cre lines target β-cell expression, and both models showed defects in *in vivo* and *in vitro* glucose-stimulated insulin secretion, reduced serum insulin levels, and impaired glucose tolerance. A companion tamoxifen-inducible βCKO mice was made using Ins1-CreERT2, and these animals phenocopy the constitutive cilia-knockout βCKO mice in defective first-phase insulin secretion, which in βCKO mice is characterized as being accompanied by delayed and reduced calcium entry into the cell (18). These results suggest a potential hierarchical and temporal regulation between primary cilia activation, β-cell membrane depolarization, and insulin release, and it would be interesting to characterize spatiotemporal relationships between β-cell ciliary and cytosolic [Ca^2+^], a line of investigation that may clarify the cilia-dependent intracellular events during GSIS.

Intriguingly, Pdx1-driven βICKO islets also revealed a defect in EphA/ephrin-A signaling, a juxtacrine pathway known to regulate insulin secretion (68). Loss of β-cell cilia was associated with EphA3 receptor hyperphosphorylation and blunted glucose-stimulated insulin secretion, rescuable by EphA5/EphrinA5 antagonism (17). Correspondingly, diabetic human islets as well as IFT88 knockdown in human islets also demonstrated EphA3 hyperphosphorylation and defective GSIS (17). In the Ins1Cre-driven βCKO cilia knockout mouse islets, however, the use of Eph/ephrin-A5-Fc modulated insulin and glucagon secretion levels to similar degrees as in normal control islets, suggesting no change in sensitivity to juxtacrine modulators (18). Despite these discrepant findings which may have been attributable to potentially different efficiencies and specificities of the Cre lines used (69–72) and how the pharmacologic studies were performed, the βICKO and βCKO models show strong agreement that IFT and cilia loss causes abnormalities in glucose-stimulated insulin secretion that may contribute to the development of metabolic diseases such as type 2 diabetes. To-date, these two β-cell IFT88 knockout mouse lines are the best models for studying cilia regulation of insulin secretion and glucose homeostasis, as these animals are viable through adulthood, and the metabolic consequences of β-cell cilia loss can be examined in mature islets without confounding exocrine abnormalities.

Given that β-cells are regulated not only by glucose but also by paracrine cues in the local islet environment, an additional role of cilia in hormonal paracrine regulation was uncovered in the βCKO mouse model (18). Primary cilia in islet β-cells express both the insulin receptor and somatostatin receptor 3 SSTR3 (16, 73, 74). The ciliary localization of these receptors suggest that the cilia may regulate β-cell responses to autocrine and paracrine signals in the islet microenvironment. Consistent with this notion, glucagon and somatostatin secretion in β-cell cilia knockout islets were higher under basal low glucose conditions, and their dynamic secretion were disrupted in the high glucose condition, suggesting impaired paracrine sensitivity by the β-cell and compensatory increases in hormone secretion by α- and δ-cells. Correspondingly, insulin suppression by exogenous somatostatin was ablated in βCKO islets, showing that cilia are required to mediate β-cell responsiveness to somatostatin (18). Thus, when β-cells lose their cilia, they lose the ability to respond to extracellular glucose as well as to paracrine signals produced by neighboring islet cells. Collectively, these studies suggest that IFT88 and primary cilia play a crucial role in islet hormone cross-regulation (18).

## Open Questions in Islet Cilia Research

Thusfar, we discuss established experimental models in the study of primary cilia, ranging from whole-body to whole-pancreas to β-cell-specific knockouts of multiple cilia genes. Systemic cilia deletion in mice results in mixed metabolic defects due to cilia dysfunction in multiple organs in the body, which complicate interpretation of their phenotypes (8, 9, 12, 16). The clearest findings to-date come from β-cell specific cilia knockouts using the highly efficient Ins1-Cre or Pdx1-Cre, particularly in the inducible setting (17, 18), which demonstrate a conserved role of cilia in insulin secretion and *in vivo* glucose homeostasis. The regulation of islet β-cells by their cilia goes beyond cell-autonomous processes such as GSIS, as cilia also mediate β/δ-cell paracrine crosstalk and potentially bidirectional signaling *via* Eph-ephrins and cell-cell contact (17, 18). As the islet field moves toward an integrated understanding of cellular connectivity and continues to build on tri-partite hormone regulation models (75), studies on islet cell cilia should aim to elucidate both cell-intrinsic ciliary pathways regulating glucose-stimulated hormone secretion, as well as cilia-mediated juxtacrine and paracrine signaling among different cell types. Therefore, we propose several obvious “next-step” lines of investigation for studying cilia function in islets.

## α- and δ-Cell Cilia

**Figure 1:**
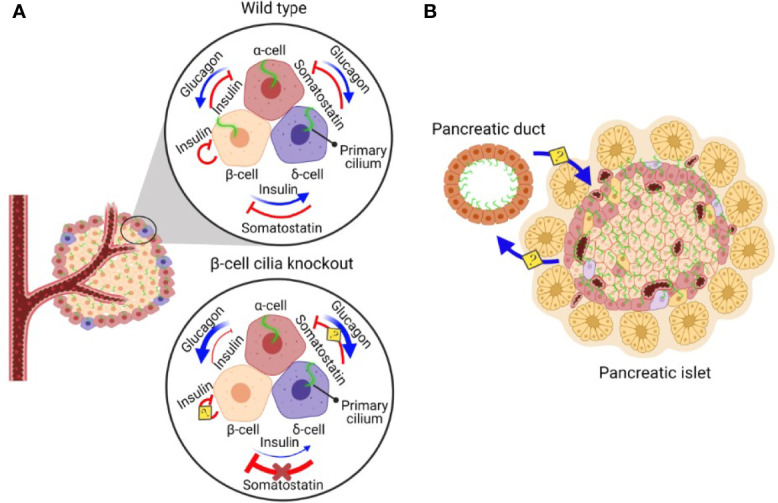
A schematic diagram of β-cell cilia-modulated islet hormone regulation and putative reciprocal communication between pancreatic ductal and islet cilia. **(A)** In normal pancreatic islets, α-cells secrete glucagon, stimulating insulin and somatostatin secretion. β-cells secrete insulin, inhibiting glucagon secretion and stimulating somatostatin secretion. δ-cells secrete somatostatin, inhibiting both glucagon and insulin secretion (73). In pancreatic islets without β-cell cilia, the ability of β-cells to regulate α- and δ-cells *via* insulin secretion is disrupted, leading to dysregulated hormone secretion from α- and δ-cells. Inhibitory somatostatin effects on β-cells are lost in the absence of β-cell cilia, leading to somatostatin insensitivity and driving compensatory increases in somatostatin secretion by δ-cells (18). The role of cilia in insulin-mediated inhibitory autocrine signalling and somatostatin-inhibited glucagon secretion await clarification using cell-specific cilia deletion models. **(B)** Pancreatic ducts are ciliated and in close proximity to the cilia on pancreatic islet cells, raising the possibility that pancreatic ductal cilia may reciprocally communicate with islet cell cilia to regulate differentiation, proliferation, and function of the pancreatic endocrine and exocrine compartments.

In addition to β-cells, primary cilia also exist on α- and δ-cells (76), and it has been demonstrated that α-cell cilia contain unique GPCRs that regulate glucagon secretion (77). It is likely that the cilia of all three cell types harbor some combination of insulin/glucagon/somatostatin hormone receptors to mediate paracrine signaling. Given the dense cytoarchitecture of islets where α-, β-, and δ-cell bodies and their cilia are in close opposition (37), primary cilia may be a key physical structure that mediates reciprocal signaling among adjacent α-, β-, and δ-cells (**Figure 1A**). An α-cell-specific cilia knockout model would be particularly useful for studying cilia regulation of glucagon secretion, as a potential therapeutic strategy for targeting hyperglucagonemia in human diabetes. Extrapolating from β-cell cilia knockout models, cilia deletion in α-cells would be expected to produce strong defects in glucagon secretion and α-cell responses to the paracrine hormones insulin and somatostatin, as well as indirectly impaired secretion of the latter two hormones. Loss of islet cell cilia or ciliary function would likely produce cell type-dependent effects on cell cycle regulation and maintenance of α-, β-, or δ-cell mass or identity, as it has been demonstrated in the exocrine pancreas where deletion of primary cilia in Kif3a-null mice induces ductal proliferation but metaplasia and apoptosis of acinar cells (12). Thus, another unexplored area would be to identify the trophic or inhibitory signals that cilia provide for the maintenance of islet α/β/δ-cell mass, either for the cell it resides on or in neighboring cell populations. Finally, as we move toward finer understanding of ciliary action in islet cells, future experimental models should aim to modulate ciliary function rather than ablating the entire ciliary structure, which would impact multiple cilia-dependent signaling pathways and processes that may be crucial for cellular survival and function.

## Non-Endocrine Cilia

In addition to endocrine cell cilia, non-endocrine cells also possess primary cilia which may play a role in regulating islet homeostasis. Pancreatic ductal cells are abundantly ciliated, while cilia and ciliary regulation of cell proliferation become lost in pancreatic cancers such as pancreatic ductal adenocarcinoma cancer (PDAC) (26, 78). Endocrine function is often disrupted in these cancers, suggesting deleterious exocrine-to-endocrine signaling, while chronic hyperglycemia in diabetes are considered risk factors for PDAC (79–82). Thus, there likely exists bidirectional exocrine-endocrine crosstalk amidst a shared milieu of pro-inflammatory and growth signals, including insulin itself, that may drive both neoplastic transformation and β-cell dysfunction in diabetes. There have been recent demonstrations of such bidirectional communication between the exocrine and endocrine pancreas; islet cell stress in diabetes has been shown to promote exocrine acinar cell ER stress, while abnormal exocrine to β-cell crosstalk causes β-cell dysfunction and loss in human diabetes (83–86). Therefore, it would be of interest to examine potential reciprocal communication between pancreatic epithelial and islet cilia (**Figure 1B**). As an example, acinar cell clusters at the exocrine-endocrine border have been shown to promote human islet cell replication through secreted REG1α proteins (87). These exocrine cells are in intimate contact with α- and β-cells through a common capsule of continuous basement membranes and extracellular matrix (ECM), and swapping of secretory vesicles has been observed between adjacent cells (87). It remains to be examined whether primary cilia or other specialized subcellular compartments play a role in either sending or receiving these exchanges. Moreover, disrupted microenvironments that affect these cellular exchanges, such as when the physical contact between islet cells and their cilia are altered by inflammation or autoimmunity, may impair β-cell function in both human type 1 and type 2 diabetes.

The islet microvasculature is richly interactive with β-cells, which are arranged in polarized contacts with the capillaries to optimize glucose sensing and insulin granule exocytosis (36, 88–91). Intriguingly, islet endothelial cells also possess cilia, which have been shown to regulate vascular function and glucose metabolism (72). Correspondingly, Bbs4 mutant islets with their intact cilia but defective ciliary/basal body function demonstrate delayed revascularization during islet engraftment, impaired vascular barrier function and glucose homeostasis, and disrupted VEGFA-VEGFR2 signaling pathway (72). These findings suggest potential communication and reciprocal regulation between islet cell and endothelial cell cilia, a theme that may be extended to other non-endocrine cell types of the islet. The growing number of single cell RNA-seq studies in human islets should make it feasible to not only analyze expression of cilia genes in these disparate cell types, but also to define the transcriptional signature associated with cilia perturbation in different pancreatic compartments and to identify cilia gene changes in human diseases such as type 2 diabetes.

## Ciliary Signaling

The cilia-centrosome complex are important regulators of cellular signaling. Primary cilia signaling pathways in pancreatic development and disease have been well-studied and well-reviewed, including Hedgehog (Hh), Wnt, Notch, and TGF-β (76). Among these, Gli/Hedgehog signaling is strongly linked to cilia and is shown to regulate homeostasis of both the endocrine and endocrine pancreas (92). Recent findings have also highlighted the importance of ciliary G protein-coupled receptor (GPCR) signaling in metabolism which, in islet cells, are exemplified by the free fatty acid receptor 4 (FFAR4/GPR120) and prostaglandin E receptor 4 (PTGER4) (77). These metabolically important GPCRs are localized in α- and β-cell primary cilia, are distinctly druggable, and represent attractive targets for pharmacologic manipulation. FFAR4 and PTGER4 agonists have been shown to promote ciliary cyclic AMP (cAMP) signaling and stimulate glucagon and insulin secretion in mouse and human islets *via* TULP3-assisted receptor trafficking (77, 93). GPR120 is also an important GPCR on δ-cells which has been shown to regulate somatostatin secretion from mouse islets (94), and activation of GPR120 signaling in δ-cells stimulates glucagon and insulin secretion (95), though it is not known whether this receptor functions through the δ cell primary cilia. The localization or recruitment of signaling receptors to cilia and direct visualization of downstream signaling e.g. cAMP, would lend strong support that these are *bona fide* ciliary signaling pathways. Other than peptide hormones and GPCR ligands, cilia also signal through a variety of growth factors, morphogens, and through the release of bioactive vesicles called ectosomes (96, 97). As proteomic methods become more accessible for cilia research, it would be informative to examine the ciliary proteome or secretome on α/β/δ cells to reveal additional pathways and other bi-directional signaling mechanisms that regulate islet cell function.

The signaling capacity of primary cilia is likely vast and context-dependent, relying on physical proximity to signaling partners in a compact tissue system such as pancreatic islets. For α/β/δ cells, ciliary signaling may be autocrine, juxtacrine, paracrine, or endocrine in nature, mirroring the myriad interactions the cilium may have with its surrounding structures (**Figure 1**). While autocrine signaling *via* cilia has not been formally studied in islets, the observation of dynamic ciliary recruitment of IR A in β-cells (16) suggest a possible role of secreted insulin feeding back onto the cell to modulate further secretion. In an analogous example of physical islet cell crosstalk *via* cellular extensions, δ-cells spread their filopodia toward other endocrine cells to form dynamic interactions and contacts, allowing the δ-cells to reach a large number of neighbors and thus increasing the efficiency of paracrine regulation (98). Likewise, the long and flexible structure of primary cilia allows them to occupy narrow spaces between islet cells, and their putative motility deriving from non-”9+0” microtubule arrangements would serve to amplify both adjacent and non-adjacent cell interactions. These dynamic cilia behaviors would add a physical dimension in islet cellular crosstalk, and studying them *in situ* would benefit from future development of cilia reporter models amenable for live-cell or intravital microscopy, and in isolated human islets, more reliable cilia biosensors and methods of manipulating endogenous ciliary gene expression. Given the heterogeneity and higher degree of intermingling of α-, β-, and δ-cells in human islets (99–101), primary cilia likely play an even more prominent role in human than mouse islets in coordinating heterotypic cell-cell interactions and maintaining functional integrity of the islet as a unit.

## Concluding Remarks

There has been much advance in understanding the role of primary cilia in islet function since their ultrastructural description more than 60 years ago. Emerging consensus from experimental models and targeted genetic knockouts reveals β-cell-specific mechanisms of cilia-dependent insulin secretion and paracrine/juxtacrine communication. Future studies combining new genetic models, molecular imaging techniques, and proteome/transcriptome analyses will bring to light additional roles of the primary cilium in the islet microenvironment, including GPCR signaling and dynamic cilia-cell and cilia-cilia interactions, and the discovery of gene signatures that may reveal potential ciliary targets to treat human type 2 diabetes and ciliopathy-related metabolic diseases.

## Author Contributions

JHC and JWH co-wrote the review article. Table and Figure are by JHC. Both authors approved the submitted version of the article.

## Funding

This work was supported by the National Institutes of Health grants DK115795A and DK127748 to JWH.

## Conflict of Interest

The authors declare that the research was conducted in the absence of any commercial or financial relationships that could be construed as a potential conflict of interest.

## Publisher’s Note

All claims expressed in this article are solely those of the authors and do not necessarily represent those of their affiliated organizations, or those of the publisher, the editors and the reviewers. Any product that may be evaluated in this article, or claim that may be made by its manufacturer, is not guaranteed or endorsed by the publisher.
